# Effects of Vibration Training on Bone Mineral Density and Balance in Postmenopausal Women with Osteopenia: A Systematic Review with Meta-Analysis

**DOI:** 10.3390/jcm15218362

**Published:** 2026-10-27

**Authors:** Jordan Hernandez-Martínez, Izham Cid-Calfucura, Edgar Vásquez-Carrasco, Tomás Herrera-Valenzuela, Pedro Delgado-Floody, Pablo Valdés-Badilla

**Affiliations:** 1 Department of Physical Activity Sciences, Universidad de Los Lagos, Osorno 5290000, Chile; jordan.hernandez@ulagos.cl; 2 Department of Education, Faculty of Humanities, Universidad de La Serena, La Serena 1700000, Chile; 3 Department of Physical Activity, Sports and Health Sciences, Faculty of Medical Sciences, Universidad de Santiago de Chile (USACH), Santiago 8370003, Chile; izham.cid@gmail.com (I.C.-C.); tomas.herrera@usach.cl (T.H.-V.); 4 School of Occupational Therapy, Faculty of Psychology, Universidad de Talca, Talca 3465548, Chile; edgar.vasquez@utalca.cl; 5 Centro de Investigación en Ciencias Cognitivas, Faculty of Psychology, Universidad de Talca, Talca 3465548, Chile; 6 VITALIS Longevity Center, Universidad de Talca, Talca 3465548, Chile; 7 Department of Physical Education, Sport and Recreation, Universidad de La Frontera, Temuco 4811230, Chile; pedro.delgado@ufrontera.cl; 8 Department of Physical Activity Sciences, Faculty of Education Sciences, Universidad Católica del Maule, Talca 3530000, Chile; 9 Sports Coach Career, Faculty of Life Sciences, Universidad Viña del Mar, Viña del Mar 2520000, Chile

**Keywords:** environmental health, bone and bones, body composition, frail elderly, exercise therapy

## Abstract

**Background:** Osteopenia is highly prevalent after menopause and increases the risk of osteoporosis, falls, and fragility fractures. Whole-body vibration has been proposed as a low-impact exercise strategy, but its clinical effectiveness remains uncertain. This systematic review with meta-analysis aims to analyze the effects of whole-body vibration on bone mineral density and balance in postmenopausal women with osteopenia. **Methods:** Five electronic databases were searched following PRISMA guidelines. Randomized controlled trials including postmenopausal women diagnosed with osteopenia by dual-energy X-ray absorptiometry were included. Methodological quality, risk of bias, and certainty of evidence were assessed using TESTEX, RoB 2, and GRADE, respectively. Effect sizes were calculated using Hedges’ g with 95% confidence intervals. **Results:** Five randomized controlled trials involving 248 women were included. Interventions lasted from 8 weeks to 12 months. Whole-body vibration did not produce significant improvements in bone mineral density at the tibia, radius, or lumbar spine, nor did it significantly improve balance compared with control conditions. The certainty of evidence was low for bone mineral density and very low for balance, mainly due to risk of bias, small sample sizes, and imprecision. **Conclusions:** Current evidence does not provide sufficient support for clinically meaningful improvements in BMD or balance following WBV in postmenopausal women with osteopenia. Nevertheless, conclusions remain uncertain because they are based on only five randomized controlled trials and very few studies per pooled outcome. Therefore, WBV cannot currently be recommended as a stand-alone intervention for improving BMD or balance, but neither can a potentially beneficial effect be definitively excluded. Larger, adequately powered, and methodologically rigorous trials are required before firm clinical recommendations can be established.

## 1. Introduction

Bone mineral density (BMD) progressively declines with aging, and this process accelerates in women during the menopausal transition because of the reduction in circulating estrogen and the resulting imbalance between bone formation and resorption [1,2]. Consequently, osteopenia represents a highly prevalent skeletal condition among postmenopausal women and constitutes an important intermediate stage between normal bone mass and osteoporosis. Population-based studies using dual-energy X-ray absorptiometry have reported osteopenia in approximately 40–50% of women aged 50 years or older, although prevalence estimates vary according to age, ethnicity, skeletal site, and diagnostic criteria [3,4]. The public health burden of osteopenia should not be underestimated: although the fracture risk of an individual woman with osteopenia is lower than that of a woman with osteoporosis, the substantially larger number of women within the osteopenia range means that a considerable proportion of fragility fractures occurs in this population. In a population-based cohort, 48.0% of older women were classified as having osteopenia, and 56.5% of all incident fractures occurred among women within the osteopenia range [5]. Moreover, the burden of low BMD among postmenopausal women is increasing alongside global population aging and is responsible for a substantial proportion of bone-related disability and mortality in women [6]. Therefore, early identification and implementation of preventive strategies including adequate calcium and vitamin D intake, regular weight-bearing and strength exercise, and interventions targeting balance and fall risk are essential to reduce progression to osteoporosis and the subsequent burden of fragility fractures [3,4,5,6].

Leading an active lifestyle through regular physical activity has been shown to have positive effects on the treatment of osteopenia and the prevention of osteoporosis [7,8]. A study conducted by Kotsili et al. [9] on postmenopausal women with osteopenia found that a 4-month intervention involving multicomponent training significantly improved cardiorespiratory fitness, balance, and BMD in the lumbar spine and total hip compared to a physically inactive control group. However, in a study conducted by Kemmler et al. [10] involving older people with osteopenia who underwent a 12-month strength training intervention, no significant increases in total hip BMD were reported; only maximum hip/leg extensor strength increased compared to a physically inactive control group. Given that the evidence regarding conventional physical exercise is inconclusive, alternatives such as whole-body vibration (WBV) training are being proposed; this approach has been shown to have high adherence and a lower injury risk compared to conventional physical exercise [11,12], with a positive clinical impact on postmenopausal women with osteopenia [11,12]. In a network meta-analysis conducted by Ma, Su and Liu [11] on postmenopausal women, improvements in femoral neck BMD were observed, with concurrent training showing a 4.9% increase, WBV 2.6%, strength training 1.8%, aerobic training 1.5%, and mild exercise 0.9%. These interventions were associated with a lower injury risk and higher adherence compared to the other interventions. In the umbrella review conducted by Yin et al. [13], which included 15 systematic reviews, 12 of which incorporated meta-analyses, no significant effects favoring WBV on BMD in postmenopausal women were reported. However, 13 of the included reviews were rated as having critically low methodological quality, and eight presented a high risk of bias. While it was suggested that WBV might have a potential role in maintaining BMD or attenuating bone loss, the authors concluded that the available evidence was insufficient to establish definitive benefits or to support its use specifically for increasing BMD.

To our knowledge, this is the first systematic review and meta-analysis specifically restricted to randomized controlled trials involving postmenopausal women diagnosed with osteopenia, while simultaneously synthesizing the effects of WBV on both BMD and balance. In addition, this review incorporates contemporary methodological standards, including TESTEX for exercise-specific methodological quality, RoB 2 for risk of bias, and GRADE for certainty of evidence, thereby providing a more rigorous and clinically relevant appraisal of the current evidence. Therefore, this systematic review and meta-analysis aims to evaluate the effects of WBV on primary outcomes (BMD) and secondary outcomes (balance) in postmenopausal women with osteopenia.

## 2. Methods

The PRISMA guidelines were adhered to in this systematic review with meta-analysis [14]; PROSPERO (the International Prospective Register of Systematic Reviews; ID code: CRD420261394770) has the protocol registered. The methodology of this systematic review and meta-analysis was conducted in accordance with the *Cochrane Handbook for Systematic Reviews of Interventions* (version 6.5), together with the PRISMA 2020 statement (Supplementary Material Table S1), whenever applicable [15].

### 2.1. Eligibility Criteria

Original peer-reviewed studies published up to June 2026 were considered without restrictions regarding publication language or publication date. Conference abstracts, books, book chapters, editorials, letters to the editor, study protocols, review articles, case reports, and non-randomized or uncontrolled studies were excluded. Eligibility was established according to the Population, Intervention, Comparator, Outcomes, and Study Design (PICOS) framework. The detailed eligibility criteria are presented in Table 1.

### 2.2. Information Search Process and Databases

The search process was conducted between July 2025 and June 2026 using five generic databases: PubMed, Medline, CINAHL Complete, Scopus, and Web of Science (core collection). The US National Library of Medicine Medical Subject Headings (MeSH) used free language terms related to Vibration Training on BMD and Balance in Postmenopausal Women with Osteopenia. The search string used was as follows: (“osteopenia” OR “osteopenic”) AND (“whole-body vibration” OR “whole body vibration” OR “vibration training” OR “vibratory training” OR “vibration exercise” OR “vibratory exercise” OR “vibration therapy” OR “WBV”) AND (“bone mineral density” OR “bone density” OR “BMD” OR “bone mass” OR “bone health” OR “balance” OR “postural balance” OR “postural control”) AND (“women” OR “woman” OR “female” OR “females” OR “postmenopause” OR “postmenopausal” OR “post-menopause” OR “post-menopausal”). To assist in locating additional relevant studies, two independent experts were consulted on the included publications and the inclusion and exclusion criteria. We stipulated two requirements for the experts: (i) to hold a PhD in sport science and (ii) to have peer-reviewed publications on physical performance in various population groups and/or physical performance published in journals with an impact factor according to Journal Citation Reports^®^. We did not disclose our search strategy to specialists to avoid bias in their searches. After completing these steps, we searched a database on 15 June 2026 for relevant retractions or errata related to the listed papers.

### 2.3. Studies Selection and Data Collection Process

The EndNote reference manager (version X9, Clarivate Analytics, Philadelphia, PA, USA) exported the studies. J.H.M. and I.C.C. conducted separate searches, eliminated duplicates, examined titles and abstracts, and examined complete texts. At this point, no disparities were discovered. The procedure was repeated for recommendations made by outside specialists and searches inside reference lists. The texts of possibly suitable papers were then examined, and the rationale behind excluding those not fitting the selection criteria was disclosed.

### 2.4. Methodological Quality Assessment

The methodological quality of the included studies was assessed using the Tool for the Assessment of Study Quality and Reporting in Exercise (TESTEX), a validated instrument specifically developed for exercise training trials [16]. TESTEX comprises 15 points, including five items evaluating study methodological quality (e.g., eligibility criteria, randomization, allocation concealment, baseline group similarity, and assessor blinding) and ten items evaluating the completeness of exercise intervention reporting (e.g., outcome assessment, intention-to-treat analysis, between-group statistical comparisons, reporting of variability, activity monitoring in the control group, exercise intensity, exercise volume and energy expenditure, adherence, and adverse events) [16]. Consistent with previous systematic reviews, studies were not excluded based on their TESTEX score; instead, the tool was used to characterize methodological quality and facilitate interpretation of the evidence. Two authors (J.H.M. and I.C.C.) independently performed the assessment, while disagreements were resolved through discussion with a third reviewer (T.H.V.). When consensus could not be reached, a fourth reviewer (P.V.B.) made the final decision.

### 2.5. Data Synthesis

The following data were obtained and analyzed from the selected studies: (i) author and year of publication; (ii) country; (iii) sex and mean ages; (iv) groups and sample size; (v) body composition assessments; (vi) diagnostic criteria for osteopenia; (vii) dosage training (total duration, weekly frequency and time per session); (viii) training intensity; (ix) primary outcome (BMD); (x) secondary outcome (balance); (xi) adverse events and adherence.

### 2.6. Risk of Bias in Individual Studies

Two independent researchers (J.H.M. and I.C.C.) evaluated the risk of bias version 2 (RoB 2) of the included studies, and a third researcher (P.V.B.) analyzed the results. The *Cochrane Handbook for Systematic Reviews of Interventions*’ recommendations for RCTs was the foundation for this evaluation [17]. Based on the randomization procedure, departures from the planned interventions, missing outcome data, outcome assessment, and choice of the reported result, the risk of bias was categorized as “high,” “low,” or “some concerns.”

### 2.7. Summary Measures for Meta-Analysis

The study methodology includes meta-analysis; full information is available in PROSPERO (registration code: CRD420261394770). Meta-analyses were only performed in the present case when ≥2 studies were available [18]. Effect sizes (ESs; Hedges’ g) for each BMD and balance WBV and control groups (CGs) were calculated using the pretraining and post-training mean and standard deviation (SD) for each dependent variable. Data were standardized using the change score SD. The ES values are presented with 95% confidence intervals (95%CIs). The calculated ES was interpreted using the following scale: trivial: <0.2; small: 0.2–0.6; moderate: >0.6–1.2; large: >1.2–2.0; very large: >2.0–4.0; extremely large: >4.0 [19]. The random effects model was used to account for differences between studies that might affect the effect of WBV. Comprehensive meta-analysis software (Version 2.0; Biostat, Englewood, NJ, USA) was used. Statistical significance was set at *p* ≤ 0.05 [20] and was used to perform these calculations. In each trial, the random effects model (DerSimonian–Laird approach) was used to calculate and pool the SMD and MD of BMD and balance (WBV vs. CG). The fundamental premise of the random effects model is that genuine effects (interventions, duration, among others) vary throughout studies and that samples are selected from populations with varying effect sizes.

Heterogeneity between trial results was tested with a Cochran’s Q test [21] and I^2^ statistic. I^2^ values of <25%, 25–50%, and >50% represent small, medium, and large amounts of inconsistency [22]. Small-study effects and potential publication bias were planned to be assessed using Egger’s regression test when at least 10 studies were available for a given meta-analysis. A two-sided *p*-value < 0.10 was considered indicative of statistically significant funnel plot asymmetry. When fewer than 10 studies were available, Egger’s regression test was not performed because of its limited statistical power and the increased risk of misleading results [23]. Funnel plots were interpreted cautiously and used only as a visual exploratory tool because asymmetry may arise from factors other than publication bias, including between-study heterogeneity, methodological differences, and sampling variability.

Sensitivity analyses were conducted to evaluate the robustness of the multilevel meta-analysis. First, a study-level leave-one-out analysis was performed by sequentially excluding each study and refitting the multilevel random effects model. Second, influence diagnostics, including Cook’s distance, DFBETAS, hat values, covariance ratios, and studentized deleted residuals, were examined to identify potentially influential studies. Finally, subgroup analyses according to comparator type (active vs. inactive controls) were conducted to explore the impact of clinical heterogeneity.

### 2.8. Certainty of Evidence

Studies were assessed for certainty of evidence using the Grading of Recommendations, Assessment, Development, and Evaluation (GRADE) scale [24] and classified as having high, moderate, low, or very low certainty of evidence. All analyses started with a high degree of certainty due to the inclusion of studies with an experimental design (randomized controlled trial and non-randomized controlled trial) and were downgraded if there were concerns about the risk of bias, consistency, accuracy, precision, directness of results, or risk of publication bias [24]. Two authors (J.H.M., I.C.C.) assessed the studies independently, and any discrepancies were resolved by consensus with a third author (P.V.B.).

### 2.9. Use of Generative Artificial Intelligence

Generative artificial intelligence (ChatGPT, OpenAI, San Francisco, CA, USA) was used exclusively to assist in the conceptual design and visual layout of the clinical implications figure. The scientific content, interpretation, figure organization, terminology, and final verification were performed entirely by the authors, who assume full responsibility for the accuracy and integrity of the published material.

## 3. Results

The search process is detailed in Figure 1. In the study identification phase, a total of 146 records were found. Duplicate articles were eliminated, and studies were filtered by title, abstract and keywords, resulting in 66 references. A total of 49 articles were eliminated for not meeting the inclusion criteria. In total, 17 full-text studies were analyzed, of which three were protocol studies, seven included a population not meeting the criteria of the target population, and two contained non-quantitative/incomplete outcome data. Five articles remained for final analysis [12,25,26,27,28].

### 3.1. Study Characteristics

Five randomized controlled trials conducted in Germany, Austria, Canada, and Thailand were reported. A total of 248 postmenopausal women participated, with a mean age of 63.96 ± 6.37 years. All cases of osteopenia were diagnosed using DXA. The studies included both active CG through balance training [26,27] and inactive CG [12,25,28], with physical exercise interventions ranging from 8 weeks to 12 months. These results are presented in detail in Table 2.

### 3.2. Methodological Quality

The five studies selected [12,25,26,27,28] were equivalent to >60% of methodological quality. These results are presented in Table 3.

### 3.3. Risk of Bias

Three studies raised some concerns regarding overall risk of bias [26,27,28], whereas two studies were judged to have a high risk of bias [12,25]. The two studies rated as high risk were primarily affected by bias due to missing outcome data. At the domain level, most studies raised some concerns regarding the randomization process, all studies raised some concerns regarding deviations from the intended interventions and selection of the reported result, and most studies were judged to be at low risk for measurement of the outcome. Missing outcome data was the main domain contributing to high risk of bias in two studies. Overall, these methodological limitations indicate that the findings should be interpreted with caution. Figure 2A,B summarize the risk-of-bias assessments.

### 3.4. Meta-Analysis

No significant improvements were reported in the primary outcomes favoring WBV compared to the CG in the tibia BMD (*p* = 0.76; I^2^ = 0.0; *p* = 0.99), radius BMD (*p* = 0.76; I^2^ = 0.0; *p* = 0.98), and lumbar spine BMD (*p* = 0.40; I^2^ = 58.7; *p* = 0.59), nor in the secondary outcomes single-leg stance (*p* = 0.27; I^2^ = 13.3; *p* = 0.31). These results are presented in Figure 3 and Figure 4.

### 3.5. Sensitivity Analysis

The leave-one-ES-out sensitivity analyses showed that the overall conclusions remained unchanged after the sequential omission of each individual ES (Table 4 and Table 5). For distal tibia, the pooled estimate varied from Hedges’ g = −0.132 to 0.248, whereas for distal radius it ranged from −0.088 to 0.222. Although the direction of the effect changed after some exclusions, none of the resulting estimates reached statistical significance. The lumbar spine findings were the most stable, with estimates ranging from g = 0.140 to 0.243 and no changes in either effect direction or statistical significance. For single-leg stance, estimates ranged from g = −0.082 to 0.310; however, the direction changed depending on the comparison omitted, while statistical significance remained unchanged. Overall, no individual effect size materially altered the interpretation of the primary analyses, indicating that the absence of statistically significant effects of WBV on BMD and balance was generally robust. Nevertheless, these findings should be interpreted cautiously because the analyses were based on very few ESs, and for single-leg stance, both comparisons originated from the same study and therefore do not represent independent evidence.

### 3.6. Exploratory Multilevel Egger-Type Meta-Regression Assessing Small-Study Effects

An exploratory multilevel Egger-type meta-regression was conducted to examine potential small-study effects while accounting for the dependency among ESs derived from the same studies. For BMD, the regression coefficient was not statistically significant (β = 1.408; 95% CI: −4.645 to 7.461; *p* = 0.590), providing no evidence of an association between ES magnitude and its standard error. In contrast, a statistically significant association was observed for balance outcomes (β = 17.169; 95% CI: 9.688 to 24.650; *p* < 0.001). However, this finding was based on six dependent ESs from only two independent studies and was strongly influenced by a single large estimate. Therefore, the apparent association for balance should not be interpreted as reliable evidence of publication bias or small-study effects. Overall, the very limited number of independent studies substantially restricts the validity and interpretability of these exploratory analyses (Table 6).

### 3.7. Certainty of Evidence

According to the GRADEpro assessment, the overall certainty of evidence regarding the effect of WBV training on BMD was rated as low. This rating was based on RCTs with serious concerns related to risk of bias and imprecision, mainly due to the small number of included studies, limited sample sizes, and confidence intervals compatible with no relevant effect. No serious concerns were identified for inconsistency, as heterogeneity was low across the pooled analyses. For balance, the certainty of evidence was rated as very low because of very serious concerns related to risk of bias in performance-based outcomes and serious imprecision. Overall, the current evidence does not provide robust support for a clinically relevant effect of WBV training on BMD or balance in postmenopausal women with osteopenia; therefore, the findings should be interpreted with caution (Table 7).

## 4. Discussion

This systematic review with meta-analysis aimed to evaluate the effects of WBV on BMD and balance in postmenopausal women with osteopenia. The main finding was that WBV did not result in significant improvements in either outcome compared with CG. Although low statistical heterogeneity was observed across the pooled analyses, the overall certainty of evidence ranged from low to very low, indicating that the available evidence remains insufficient to support WBV as a stand-alone intervention for improving skeletal health or postural balance in postmenopausal women with osteopenia. Several factors may explain these findings. Specifically, the WBV protocols currently reported in the literature may not provide a mechanical stimulus of sufficient magnitude, specificity, or progression to elicit meaningful osteogenic or neuromuscular adaptations in postmenopausal women with osteopenia. Consequently, while WBV may represent an accessible exercise modality, its isolated use should be interpreted with caution when the primary therapeutic objective is to improve bone health or balance.

### 4.1. Effects of WBV on BMD

Regarding BMD, no significant improvements were observed following WBV compared with control interventions at the tibia (*p* = 0.99), radius (*p* = 0.75), or lumbar spine (*p* = 0.62). These findings are consistent with those reported by Yin, Liu, Zhong and Zhu [13] in an umbrella review that included 15 systematic reviews (three qualitative systematic reviews and 12 meta-analyses) examining the effects of WBV on BMD in postmenopausal women. Overall, the authors concluded that the current evidence does not support a significant advantage of WBV for improving BMD in this population, although this physical exercise modality may play a potential role in attenuating age-related bone loss. Some discrepancies, however, exist across the literature. Massini et al. [29], in a systematic review and meta-analysis conducted in older people, reported a small but significant effect of WBV on total femur BMD (g = 0.28; *p* = 0.04), whereas no significant improvements were observed at the femoral neck (g = 0.15; *p* = 0.19) or lumbar spine (g = 0.13; *p* = 0.31). Similarly, Ma et al. [11], in a network meta-analysis involving postmenopausal women, reported a 2.6% increase in femoral neck BMD following WBV. However, larger improvements were observed after concurrent training (4.9%) compared with strength training (1.8%), aerobic training (1.5%), and moderate-intensity exercise (0.9%). Collectively, these findings suggest that, although WBV may contribute to preserving bone health, its osteogenic potential may be lower than that of physical exercise modalities that combine weight-bearing activities with dynamic muscle contractions.

Several factors may partially explain the limited effects observed in the present systematic review with meta-analysis. First, considerable heterogeneity exists among WBV protocols, particularly regarding intervention duration, vibration frequency, and amplitude [12,25,26,27,28]. Across the included studies, the intervention duration ranged from 8 weeks to 12 months, vibration frequency from 18 to 40 Hz, and amplitude from 2 to 8 mm. This variability may be particularly relevant because bone adaptation is a slow process that depends on repeated exposure to mechanical stimuli over prolonged periods [30,31,32]. Indeed, bone tissue responds preferentially to dynamic, variable, and progressively overloaded stimuli that generate sufficient mechanical strain to activate bone formation and remodeling processes [30,32]. Therefore, some of the WBV protocols implemented may not have provided an adequate magnitude, progression, or duration of mechanical loading to induce clinically meaningful osteogenic adaptations [13].

Additionally, the estrogen deficiency associated with menopause promotes an imbalance between bone formation and resorption by increasing osteoclastic activity and reducing osteoblastic activity, which may diminish bone sensitivity to external mechanical stimuli [13,33]. Furthermore, no clear consensus currently exists regarding the optimal WBV dose for promoting bone adaptations in postmenopausal women with osteopenia [13], as substantial variability persists in the prescription of frequency, amplitude, session duration, and overall intervention length [13,33]. Taken together, these factors may partially explain the absence of significant effects observed in the present review and further support the notion that physical exercise modalities combining weight-bearing activities with dynamic muscle contractions may provide a more potent osteogenic stimulus than WBV performed in isolation [11,13].

### 4.2. Effects of WBV on Balance

No significant improvements in balance were observed following WBV compared with the CG (*p* = 0.31). However, only two studies [26,28], representing a total of three intervention groups, were included in the present meta-analysis. Therefore, these findings should be interpreted with caution due to the limited amount of available evidence. Our findings differ from those reported by Zafar et al. [34], who, in a systematic review and meta-analysis conducted in individuals with chronic low back pain, observed significant improvements in balance (*p* = 0.02) following WBV. Similarly, Liu et al. [35], in a network meta-analysis involving older people, reported significant improvements in balance (*p* < 0.05) compared with placebo and conventional rehabilitation groups. These discrepancies may be partially explained by differences in the populations studied, as well as by variations in WBV protocols and balance assessment methods across studies [34,35].

Several physiological mechanisms have been proposed to explain the potential benefits of WBV on postural control [33,36]. Vibration-induced mechanical oscillations may stimulate proprioceptors, increase afferent fiber excitability, and activate α-motor neurons, thereby eliciting reflex muscle contractions that could enhance neuromuscular coordination [33,36]. However, these physiological responses do not necessarily translate into meaningful functional improvements. This may be because balance is a multifactorial capacity that depends on the integrated contribution of visual, vestibular, somatosensory, and neuromuscular systems, and stimulation through WBV alone may be insufficient to induce clinically relevant adaptations in postural control [37,38]. Taken together, the limited number of available studies, the variability in methodological approaches, and the multifactorial nature of balance may partially explain the absence of significant effects observed in the present meta-analysis. Consequently, additional high-quality studies are required before definitive conclusions can be drawn regarding the effectiveness of WBV for improving balance in postmenopausal women with osteopenia.

### 4.3. Potential Mechanisms Explaining the Limited Effectiveness of WBV

The limited effectiveness observed in the present systematic review with meta-analysis may also be explained by age- and menopause-related alterations in bone mechanosensitivity [39,40]. Bone tissue continuously adapts to mechanical loading through the activity of osteocytes [37,41], which function as the primary mechanosensory mechanism regulating bone remodeling [32,42]. However, the hormonal changes associated with menopause [32,42,43], particularly estrogen deficiency, disrupt this adaptive process by increasing osteoclastic activity, reducing osteoblastic activity, and impairing mechanotransduction pathways involved in the conversion of mechanical stimuli into anabolic signals [40,43]. Consequently, postmenopausal women may exhibit a diminished responsiveness to external mechanical loading [44].

This altered physiological environment may be particularly relevant when interpreting the present findings, as several of the included WBV protocols may not have generated a mechanical stimulus of sufficient magnitude, specificity, or progression to counteract the accelerated bone resorption state characteristic of osteopenia [13]. Indeed, unlike physical exercise modalities that combine impact loading and dynamic muscle contractions, WBV performed in isolation may provide a relatively modest osteogenic stimulus that is insufficient to induce clinically meaningful changes in BMD [13].

Furthermore, bone adaptation is a slow and cumulative process that depends not only on the intensity of the applied stimulus but also on its progressive overload, variability, and long-term repetition [45]. Therefore, even though WBV may activate mechanosensitive pathways, the considerable variability observed across intervention protocols, together with the reduced mechanosensitivity associated with postmenopausal aging, may partially explain the absence of significant effects observed in the present meta-analysis.

Importantly, BMD should not be considered the sole therapeutic target in osteopenia management. Although BMD is a clinically relevant indicator of skeletal health, osteopenia is a multifactorial condition in which fracture risk is also influenced by declines in muscle strength, balance, mobility, and overall functional capacity [39,40]. Therefore, interventions should aim to preserve global musculoskeletal health rather than exclusively target bone density outcomes. From this perspective, physical exercise prescription should prioritize strategies capable of simultaneously improving skeletal integrity, physical function, and long-term independence in postmenopausal women [11].

Despite the limited effects observed, WBV may still offer potential advantages from an implementation perspective. Long-term adherence remains one of the greatest challenges in exercise-based interventions targeting older people and postmenopausal women [13], as barriers such as fear of falling, low exercise tolerance, mobility limitations, and reduced motivation may compromise long-term participation [46]. Because WBV is generally considered a low-impact and accessible exercise modality, it may represent a feasible option for individuals who are unable or unwilling to participate in more demanding exercise programs. However, whether WBV improves long-term exercise adherence or serves as an effective entry-point strategy for broader multicomponent exercise programs remains uncertain, as these outcomes were not systematically evaluated in the available studies. Future well-designed RCTs should investigate these implementation-related outcomes alongside traditional clinical endpoints to better define the potential role of WBV in postmenopausal women with osteopenia.

### 4.4. Clinical Implications

The present findings provide several clinical implications for healthcare professionals and sports science specialists working with postmenopausal women with osteopenia:

(i) WBV should not be considered a stand-alone strategy when the primary therapeutic objective is to improve BMD or balance, as the current evidence does not support significant benefits for these outcomes.
(ii) Based on the current evidence, WBV should not be recommended as a stand-alone intervention to improve BMD in postmenopausal women with osteopenia, as no clinically meaningful benefits were demonstrated.
(iii) Given its low-impact nature and potential feasibility in clinical settings, WBV may be considered a complementary exercise modality, especially for women with functional limitations or reduced tolerance to higher-intensity exercise interventions.
(iv) Clinicians and sports science professionals should interpret WBV prescription with caution, as considerable variability currently exists regarding vibration frequency, amplitude, intervention duration, and overall training dose, limiting the ability to establish evidence-based recommendations.
(v) Sports science professionals should prioritize multicomponent interventions combining strength training, impact exercises, and weight-bearing activities, while considering WBV as an adjunctive strategy rather than a replacement for conventional physical exercise.

These clinical implications are presented in Figure 5.

### 4.5. Strengths and Limitations

The present systematic review with meta-analysis has several limitations that should be considered when interpreting the findings. These include: (i) the relatively small number of included studies and the substantial clinical and methodological heterogeneity across them, which precluded meaningful subgroup or meta-regression analyses to determine whether training dosage (i.e., intervention duration, weekly frequency, and session duration), participant characteristics (e.g., age and body composition), differences in osteopenia diagnostic criteria, variability in BMD assessment protocols, methodological quality, or sample size influenced the observed effects; (ii) the relatively small sample sizes of the primary studies, which may have reduced the ability to detect significant effects across the analyzed outcomes; and (iii) the low and very low certainty of evidence observed for BMD and balance, respectively, indicating that future high-quality studies may modify the current estimates and provide more definitive conclusions regarding the effectiveness of WBV in postmenopausal women with osteopenia. Despite these limitations, the present review also has several strengths. These include: (i) the inclusion of studies with methodological quality scores equivalent to >60%, as assessed using TESTEX; (ii) the implementation of rigorous methodological procedures, including adherence to PRISMA guidelines, prospective registration in PROSPERO, and the use of TESTEX, RoB 2, and GRADE to evaluate methodological quality, risk of bias, and certainty of evidence; (iii) a comprehensive literature search conducted across five electronic databases (PubMed, MEDLINE, CINAHL Complete, Scopus, and Web of Science); (iv) the exclusive inclusion of randomized controlled trials, thereby increasing the methodological rigor of the synthesized evidence; and (v) the focus on a specific and clinically relevant population, namely, postmenopausal women with osteopenia, thereby reducing the clinical heterogeneity commonly observed when studies involving osteopenia and osteoporosis are analyzed together.

### 4.6. Future Perspectives and Research Directions

Future research should move beyond simply comparing WBV with an inactive CG and focus on identifying the optimal role of WBV within the management of postmenopausal osteopenia. Particular attention should be given to establishing dose–response relationships involving vibration frequency, amplitude, acceleration, session duration, weekly frequency, and overall intervention length, as considerable variability currently exists across prescription parameters. Furthermore, future RCTs should investigate the integration of WBV within multicomponent exercise programs rather than evaluating it as a stand-alone intervention. Expanding the range of clinically relevant outcomes beyond BMD may also be warranted, including muscle strength, physical function, fall risk, quality of life, and fracture incidence. Finally, future studies should determine whether specific WBV prescription parameters or combinations with other physical exercise modalities can enhance clinically meaningful outcomes related to musculoskeletal health in postmenopausal women with osteopenia.

## 5. Conclusions

This systematic review with meta-analysis found no statistically significant improvements in BMD at the tibia, radius, or lumbar spine, or in balance, following WBV compared with active or inactive control conditions in postmenopausal women with osteopenia. However, these findings are based on a very limited evidence base comprising only five RCTs and 248 participants, with only two to three studies contributing to the balance analyses and similarly small numbers informing individual BMD outcomes. In addition, the certainty of evidence ranged from low to very low because of risk of bias, small sample sizes, imprecision, and methodological heterogeneity across intervention and comparator conditions. Consequently, the absence of statistically significant effects should not be interpreted as definitive evidence that WBV is ineffective. Based on the currently available evidence, WBV cannot be recommended as a stand-alone strategy for improving BMD or balance in this population, and any clinical use should be considered cautiously and preferably as an adjunct to established multicomponent exercise approaches. Larger, adequately powered RCTs with standardized WBV protocols, clearly defined comparator groups, longer follow-up periods, and clinically relevant skeletal and functional outcomes are needed before robust conclusions and clinical recommendations can be made.

## Figures and Tables

**Figure 1:**
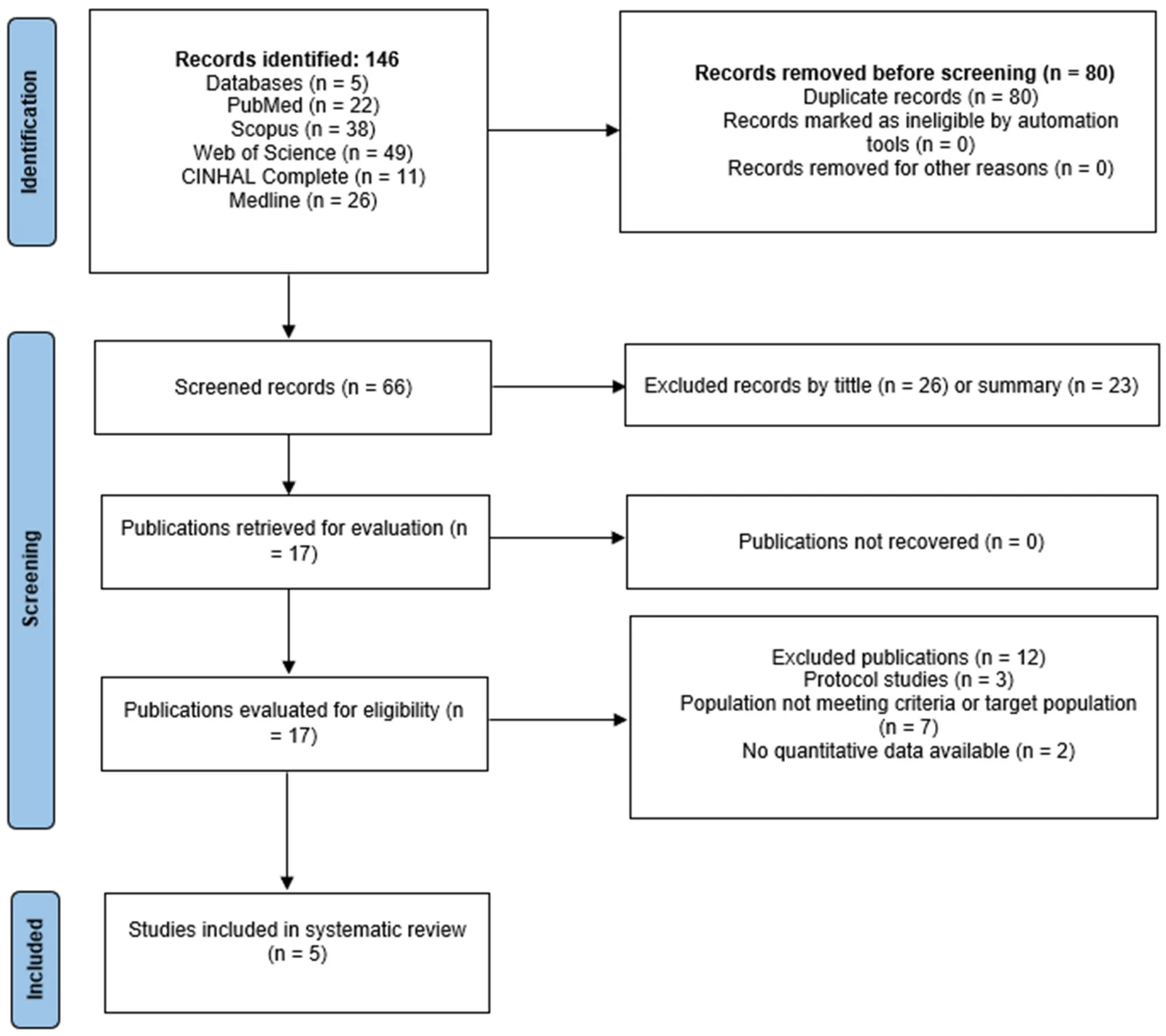
Flowchart of the review process. Based on the PRISMA 2020 guidelines [14].

**Figure 2:**
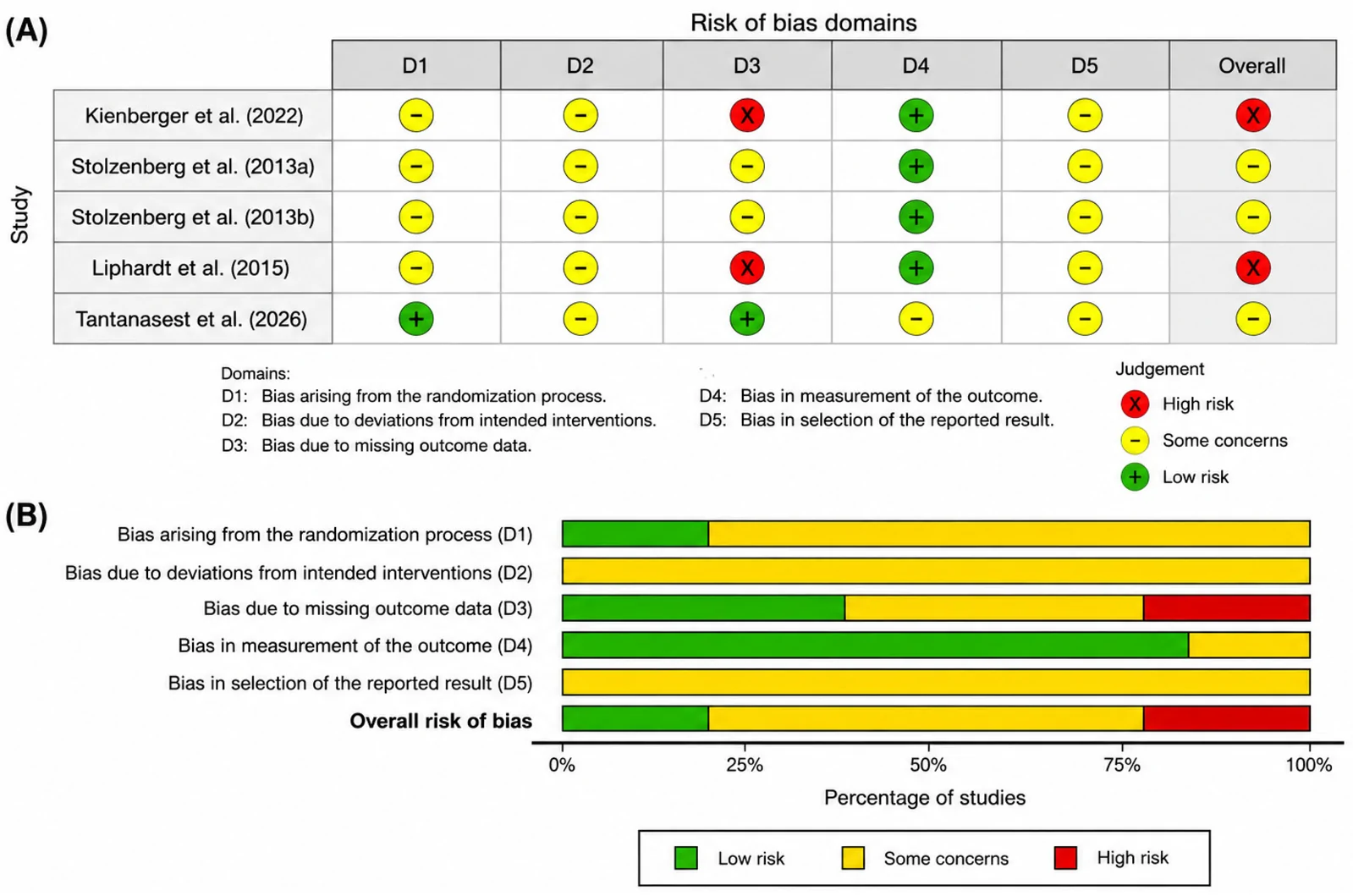
Risk of bias: (**A**) summary of risk of bias assessments; (**B**) traffic light graph [12,25,26,27,28].

**Figure 3:**
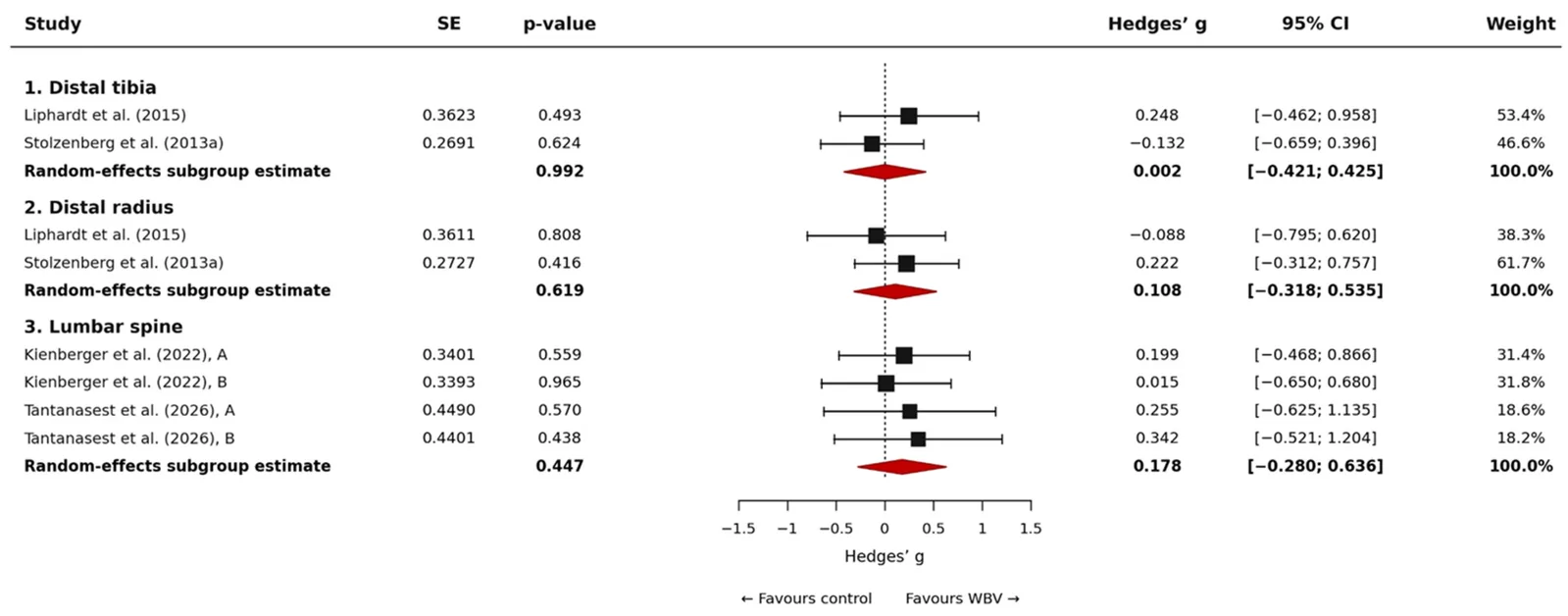
Forest plot of changes in bone mineral density with whole-body vibration training in postmenopausal women compared with postmenopausal women assigned as controls [12,25,26,28]. Values shown are effect sizes (Hedges’ g) with 95% confidence intervals (CIs). Black squares represent individual effect-size estimates, whereas red diamonds represent pooled subgroup estimates. Uppercase letters A and B identify different comparisons within the same publication. 1: Tibia; 2: Radius; 3: Lumbar spine.

**Figure 4:**
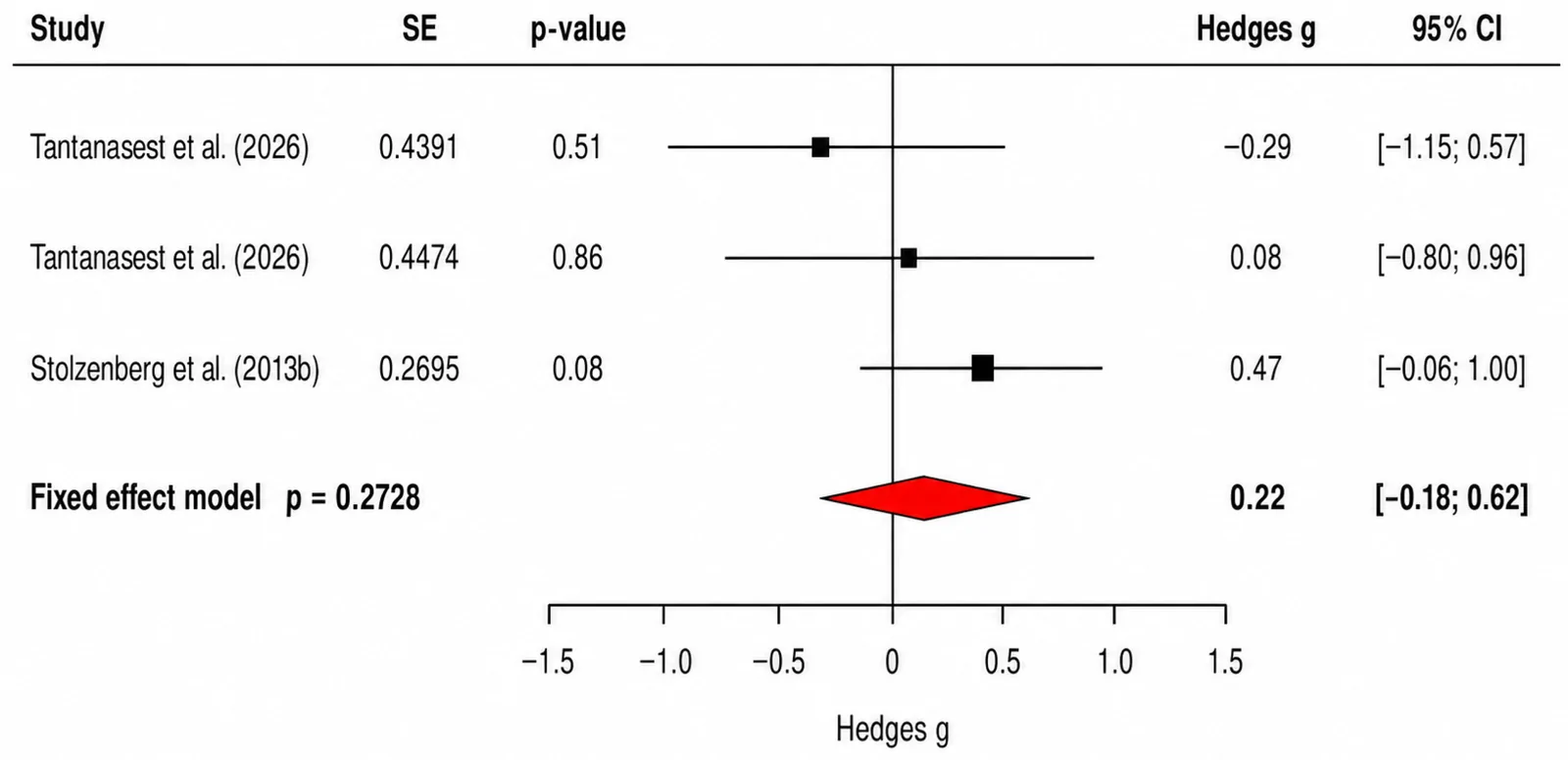
Forest plot of changes in balance with whole-body vibration training in postmenopausal women compared with postmenopausal women assigned as controls [27,28]. Values shown are effect sizes (Hedges’ g) with 95% confidence intervals (CIs). Black squares represent individual effect-size estimates, whereas the red diamond represents the pooled effect estimate.

**Figure 5:**
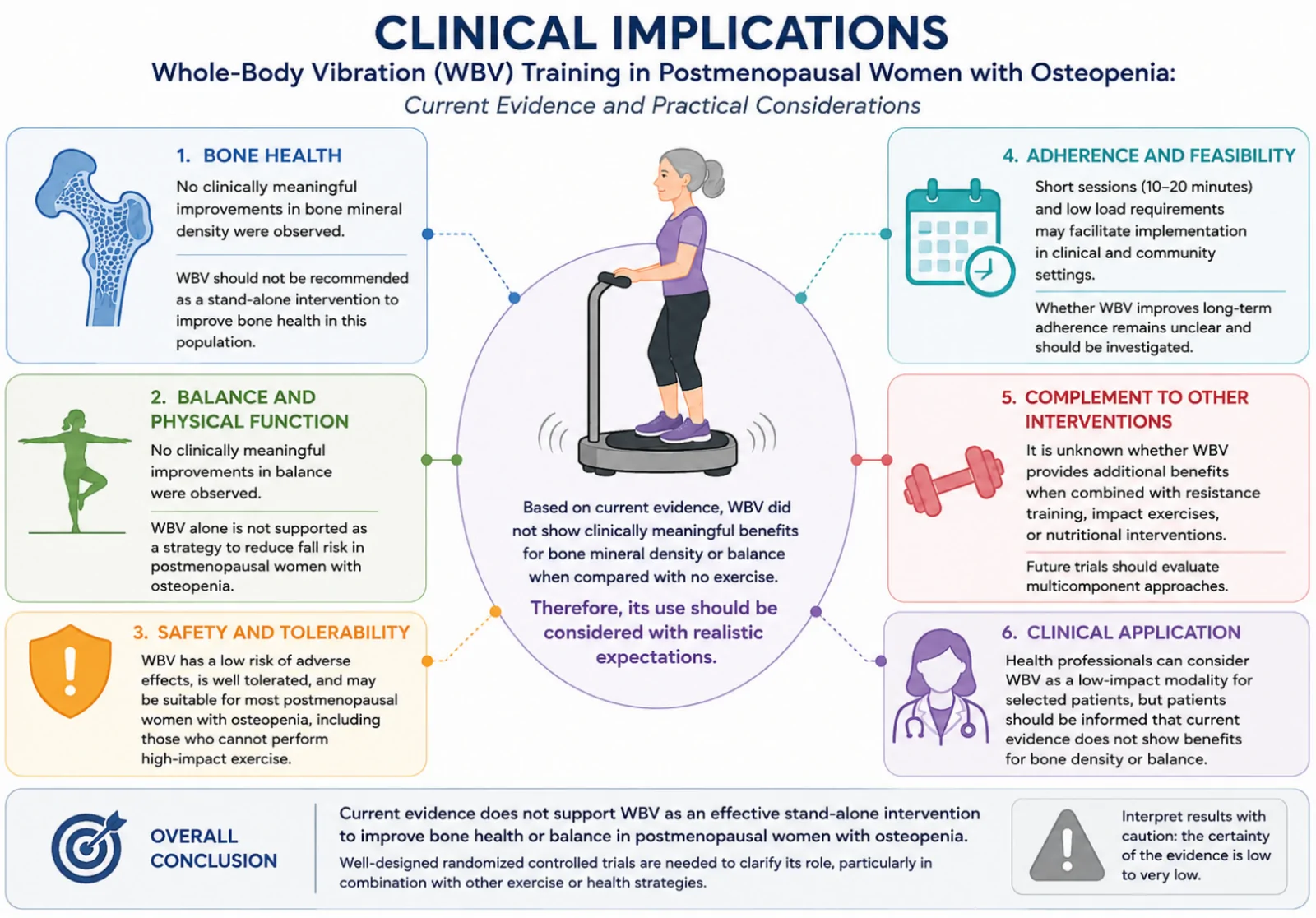
Graphical representation of the clinical implications of the reported evidence.

**Table 1:** Selection criteria used in the systematic review.

Category	Inclusion Criteria	Exclusion Criteria
Population	Postmenopausal women diagnosed with osteopenia using dual-energy X-ray absorptiometry, defined by a T-score between −1.0 and −2.5 at the femoral neck, total hip, lumbar spine, or another clinically recognized skeletal site.	Studies including mixed populations, such as men, premenopausal women, or women with osteoporosis, when data for postmenopausal women with osteopenia could not be extracted separately. Studies involving osteopenia secondary to cancer, severe metabolic bone disease, or other conditions that could substantially affect bone metabolism were also excluded.
Intervention	Whole-body vibration training delivered as the primary exercise intervention, regardless of platform type, vibration frequency, amplitude, session duration, weekly frequency, or total intervention length.	Multicomponent interventions were excluded when the independent effect of whole-body vibration could not be isolated. Studies using local vibration, focal vibration, or passive vibration applied to a single body region rather than whole-body vibration were also excluded.
Comparator	Active or inactive control conditions, including usual care, maintenance of habitual activities, balance training, resistance training, weighted-vest exercise, sham vibration, or no-exercise control groups.	Single-arm studies without a comparison group were excluded. Studies were also excluded when the experimental and comparator groups received substantially different co-interventions, preventing the specific effect of whole-body vibration from being determined.
Outcome	Primary outcomes included total or site-specific bone mineral density assessed using validated imaging methods. Secondary outcomes included direct measures of static or dynamic balance obtained through validated clinical or laboratory tests.	Studies reporting only surrogate outcomes unrelated to bone mineral density or balance, such as muscle activity, hormonal markers, pain, body composition, or quality of life, were excluded. Studies were also excluded when outcome data were insufficient for extraction or quantitative synthesis.
Study design	Randomized controlled trials.	Non-randomized controlled trials; cross-sectional, retrospective, and prospective studies.

**Table 2:** The effect of vibration training on bone mineral density and balance in postmenopausal women.

Author	Study Design	Country	Sex and Mean Age	Groups (Sample Size)	Body Composition Assessments	Diagnostic Criteria for Osteopenia	Dosage			Intensity	Primary Outcome (Bone Mineral Density)	Secondary Outcome (Balance)	Adverse Events and Adherence
							Weeks	Frequency	Minutes per Session				
Kienberger, Sassmann, Rieder, Johansson, Kässmann, Pirich, Wicker and Niebauer [12]	RCT	Austria	100% women WBV: 56.1 (5.1) RT: 62.8 (6.8) CG: 58.7 (8.2)	WBV (18): whole-body vibrative RT (22): resistance training CG (21): continue with daily activities	DXA (QDR 4500 W, Hologic, Marlborough, MA, USA).	Subjects were diagnosed as osteopenic when T-score values between −1.0 and −2.5 were measured at least at one of these sites (hip and lumbar spine).	12 months	2	NR	18 Hz to 20 Hz and 2 mm	T-score total hip left, mean differences (SD) T-score lumbar spine, mean differences (SD)	Isokinetic strength (60°/s) extensors [Nm] Isokinetic strength (60°/s) flexors [Nm] Isokinetic strength (240°/s) extensors [Nm] Isokinetic strength (240°/s) flexors [Nm] Balance test [*n*]	NR ≥85%
Liphardt, Schipilow, Hanley and Boyd [25]	RCT	Canada	100% women Total sample: 58.5 ± 3.3	WBV (17): whole-body vibrative CG (14): continue with daily activities	DXA	Women were postmenopausal and had a DXAT score of the femoral neck or lumbar spine in the osteopenic range (−1.0 to −2.5)	12 months	2–3	NR	20 Hz and 3–4 mm	Tibia Tt.Ar (mm^2^) Tt.BMD (mgHA/cm^3^) Radius Tt.Ar (mm^2^) Tt.BMD (mgHA/cm^3^) Femoral neck Lumbar spine	Knee extension torque (Nm) Maximal isometric handgrip strength (kg)	NR >90%
Stolzenberg, Belavý, Rawer and Felsenberg [26]	RCT	Germany	100% women WBV: 67.2 (3.7) BT: 66.0 (4.5)	WBV (34): whole-body vibrative BT (34): balance training	DXA (Lunar Prodigy Advance, GE Medical Systems, Wauwatosa, WI, USA)	T-score hip or lumbar spine (L1–L4) T-score from 2.0 to 3.0 SD	9 months	2	NR	22 Hz to 26 Hz and 4–8 mm	Total BMD Trabecular (BMD) Cortical (BMD) Tibia (BMD) Fibula (BMD) Radius (BMD) Ulna (BMD)	NR	Dropouts were due to injury or illness not associated with the study; No intervention-related adverse events were clearly reported ≥75%
Tantanasest, Manimmanakorn, Tunkamnerdthai, Khaengkhan, Nithisup, Youdprang, Manimmanakorn and Hamlin [28]	RCT	Thailand	100% women WBV: 67.11 ± 5.37 WV: 69.56 ± 5.61 CG: 66.00 ± 5.22	WBV (10): whole-body vibrative WV (11): weighted vest CG (10): continue with daily activities	DXA	T-score between −1.0 and −2.4 for osteopenia	8 weeks	3	40	40 Hz and 2 mm	Lumbar spine (g/cm^2^) Legs (g/cm^2^)	TUG (s) SLS (s) 5TSTS (s) 6-MWT (m)	Dropouts were due to injury or illness not associated with the study; No intervention-related adverse events were clearly reported ≥75%
Stolzenberg, Belavý, Rawer and Felsenberg [27]	RCT	Germany	100% women WBV: 65.9 (4.5) BT: 67.3 (3.7)	WBV (26): whole-body vibrative BT (31): balance training	DXA (Lunar Prodigy Advance, GE Medical Systems, Wauwatosa, WI, USA)	T-score hip or lumbar spine (L1–L4) T-score from 2.0 to 3.0 SD	9 months	2	NR	22 Hz to 26 Hz and 4–8 mm	NR	SLS (s)	NR ≥85%

Abbreviations: RCT: randomized controlled trial; WBV: whole-body vibration; RT: resistance training; BT: balance training; WV: weighted vest; CG: control group; DXA: dual-energy X-ray absorptiometry; BMD: bone mineral density; T-score: bone mineral density score expressed in standard deviations from the young adult reference mean; SD: standard deviation; NR: not reported; Hz: hertz; mm: millimeters; Nm: newton-meter; °/s: degrees per second; Tt.Ar: total bone area; Tt.BMD: total bone mineral density; mgHA/cm^3^: milligrams of hydroxyapatite per cubic centimeter; QDR: quantitative digital radiography/dual-energy X-ray absorptiometry device model; GE: General Electric; USA: United States of America; L1–L4: lumbar vertebrae 1 to 4; TUG: Timed Up and Go test; SLS: single-leg stance test; 5TSTS: five-times sit-to-stand test; 6-MWT: six-minute walk test.

**Table 3:** Study quality assessment according to the TESTEX scale.

Study	Eligibility Criteria Specified	Randomly Allocated Participants	Allocation Concealed	Groups Similar at Baseline	Assessors Blinded	Outcome Measures/Adherence/Adverse Events/Attendance *	Intention-to-Treat Analysis	Between-Group Statistical Comparisons Reported	Point Measures and Variability Reported **	Activity Monitoring in Control Group	Relative Exercise Intensity Reviewed	Exercise Volume and Energy Expenditure	Overall TESTEX #	Interpretation
Kienberger, Sassmann, Rieder, Johansson, Kässmann, Pirich, Wicker and Niebauer [12]	Yes	Yes	Yes	Yes	Yes	Yes (2)	Yes	Yes	Yes (2)	Yes	Yes	Yes	14/15	High methodological/reporting quality
Liphardt, Schipilow, Hanley and Boyd [25]	Yes	Yes	No	Yes	No	Yes (1)	No	Yes	Yes (2)	Yes	Yes	Yes	10/15	Moderate methodological/reporting quality
Stolzenberg, Belavý, Rawer and Felsenberg [26]	Yes	Yes	No	Yes	No	Yes (1)	No	Yes	Yes (2)	Yes	Yes	Yes	10/15	Moderate methodological/reporting quality
Stolzenberg, Belavý, Rawer and Felsenberg [27]	Yes	Yes	No	Yes	No	Yes (1)	No	Yes	Yes (2)	Yes	Yes	Yes	10/15	Moderate methodological/reporting quality
Tantanasest, Manimmanakorn, Tunkamnerdthai, Khaengkhan, Nithisup, Youdprang, Manimmanakorn and Hamlin [28]	Yes	Yes	No	Yes	No	Yes (2)	Yes	Yes	Yes (2)	Yes	Yes	Yes	13/15	High methodological/reporting quality

* Three points are possible: one point if adherence >85%, one point if adverse events were reported, and one point if exercise attendance was reported. ** Two points are possible: one point if the primary outcome is reported, one point if all other outcomes were reported. # total out of 15 points. TESTEX: Tool for assessing study quality and reporting in exercise.

**Table 4:** Leave-one-effect-size-out sensitivity analysis.

Outcome	Complete Model, Hedges’ g (95% CI)	*p*-Value	Effect Sizes (k)	Range After Omission	Largest Absolute Change	Most Influential Effect	Direction Changed	Significance Changed
Distal tibia	0.003 (−0.420 to 0.427)	0.988	2	−0.132 to 0.248	0.245	Stolzenberg et al. (2013a) [26], E011	Yes	No
Distal radius	0.110 (−0.317 to 0.536)	0.615	2	−0.088 to 0.222	0.197	Stolzenberg et al. (2013a) [26], E012	Yes	No
Lumbar spine	0.178 (−0.280 to 0.636)	0.447	4	0.140 to 0.243	0.065	Kienberger et al. (2022) [12], E004	No	No
Single-leg stance	0.103 (−0.650 to 0.855)	0.789	2	−0.082 to 0.310	0.207	Tantanasest et al. (2026) [28], E044	Yes	No

Note: The SLS sensitivity analysis represents leave-one-effect-out rather than leave-one-study-out because both estimates originated from the same study. Consequently, it evaluates sensitivity to individual comparisons but not robustness across independent studies. The SLS values were calculated from the rounded estimates currently available; using the original unrounded variances could produce very small numerical differences.

**Table 5:** Absolute change represents the absolute difference.

Outcome	Effect Omitted	Remaining *k*	Remaining Studies	Hedges’ *g*	95% CI	*p*-Value	Absolute Change	Direction Changed	Significance Changed
Distal tibia	Liphardt et al. (2015) [25]	1	1	−0.132	−0.659 to 0.396	0.624	0.135	Yes	No
Distal tibia	Stolzenberg et al. (2013a) [26]	1	1	0.248	−0.462 to 0.958	0.493	0.245	No	No
Distal radius	Liphardt et al. (2015) [25]	1	1	0.222	−0.312 to 0.757	0.416	0.112	No	No
Distal radius	Stolzenberg et al. (2013a) [26]	1	1	−0.088	−0.795 to 0.620	0.808	0.197	Yes	No
Lumbar spine	Kienberger et al. (2022a) [12]	3	2	0.140	−0.359 to 0.639	0.583	0.038	No	No
Lumbar spine	Kienberger et al. (2022b) [12]	3	2	0.243	−0.256 to 0.743	0.340	0.065	No	No
Lumbar spine	Tantanasest et al. (2026a) [28]	3	2	0.179	−0.300 to 0.659	0.464	0.001	No	No
Lumbar spine	Tantanasest et al. (2026b) [28]	3	2	0.151	−0.331 to 0.633	0.539	0.027	No	No
Single-leg stance	Tantanasest et al. (2026a) [28]	1	1	−0.082	−0.939 to 0.774	0.851	0.185	Yes	No
Single-leg stance	Tantanasest et al. (2026b) [28]	1	1	0.310	−0.572 to 1.192	0.491	0.207	No	No

Note: Absolute change represents the absolute difference between the complete-model estimate and the estimate obtained after omitting the indicated effect. The letters “a” and “b” identify different comparisons reported within the same publication and do not represent independent studies. Kienberger et al. (2022) [12]: a, WBV versus control; b, WBV versus resistance training. Tantanasest et al. (2026) [28]: a, WBV plus light exercise versus light exercise alone; b, WBV plus light exercise versus weighted-vest training plus light exercise. For single-leg stance, the analysis is leave-one-effect-out rather than leave-one-study-out because both comparisons come from the same study. CI, confidence interval; WBV, whole-body vibration.

**Table 6:** Exploratory multilevel Egger-type meta-regression for small-study effects.

Outcome Domain	Effect Sizes, *k*	Independent Studies, *n*	Regression Coefficient (95% CI)	*p*-Value
Bone mineral density	8	4	1.408 (−4.645 to 7.461)	0.590
Balance	6	2	17.169 (9.688 to 24.650)	<0.001

Note: The coefficient represents the association between the effect size and its standard error in an exploratory multilevel meta-regression. A within-study sampling error correlation of ρ = 0.50 was assumed. Although a statistically significant association was identified for balance, this analysis comprised six dependent effect sizes from only two independent studies and was strongly influenced by a single large estimate. Therefore, neither result provides reliable evidence for or against publication bias or small-study effects. CI, confidence interval.

**Table 7:** Evaluation of methodological quality using the GRADEpro tool.

Certainty Assessment							Number of Patients		Effect		Certainty	Importance
Number of Studies	Study Design	Risk of Bias	Inconsistency	Indirect Evidence	Imprecision	Other Considerations	[Intervention]	[Comparison]	Relative (95% CI)	Absolute (95% CI)		
Bone mineral density												
4	randomized trials	serious a	not serious	not serious	Serious a	none	79/158 (50.0%)	79/158 (50.0%)	not estimable		Moderate	Important
Balance												
3	randomized trials	very serious a	not serious	not serious	Serious a	none	54/116 (46.6%)	62/116 (53.4%)	not estimable		Low	Important

CI: confidence interval; a: some concerns.

## Data Availability

The original contributions presented in this study are included in the article. Further inquiries can be directed to the corresponding author.

## Supplementary Materials

The following supporting information can be downloaded at: https://www.mdpi.com/article/10.3390/jcm15218362/s1, Table S1: PRISMA 2020 Checklist.
